# Effects and mechanisms of *Helicobacter pylori* on cancers development and immunotherapy

**DOI:** 10.3389/fimmu.2024.1469096

**Published:** 2024-10-07

**Authors:** Xiaotian Zhong, Huiling Zheng, Shiqing Zhao, Ziye Wang, Yi Su, Kaili Zhong, Mopei Wang, Yanyan Shi

**Affiliations:** ^1^ Research Center of Clinical Epidemiology, Peking University Third Hospital, Beijing, China; ^2^ Peking University Health Science Center, Beijing, China; ^3^ Department of Gastroenterology, Peking University Third Hospital, Beijing, China; ^4^ Department of Lymphoma, Beijing Shijitan Hospital, Capital Medical University, Beijing, China; ^5^ Department of Tumor Chemotherapy and Radiation Sickness, Peking University Third Hospital, Beijing, China

**Keywords:** *Helicobacter pylori*, immunotherapy, gastric cancer, colorectal cancer, melanoma, non-small cell lung cancer

## Abstract

Tumor immunotherapy has been widely used in clinical treatment of various cancers. However, some patients of these cancers do not respond to immunotherapy effectively. And *H. pylori* infection has been considered to be related to the efficacy of immunotherapy. This review aims to summarize the different effects and mechanisms of *H. pylori* infection on immunotherapy in different kinds of cancers. We searched the relevant literature on *H. pylori* and tumor immunotherapy, and summarized to form a review. Generally, *H. pylori* infection plays a role in affecting kinds of cancers’ development, besides gastric cancer. Current evidence suggests that *H. pylori* infection may reduce the efficacy of immunotherapy for colorectal cancer, non-small cell lung cancer and melanoma, but due to the lack of sufficient evidence, more data is needed to prove that. While for gastric cancer, the effects remain controversial. The *H. pylori* regulation effects and metabolisms involved in systematic related cancers should be paid attention to. Whether *H. pylori* should be eradicated when immunotherapy performed may be a critical consideration for some kinds of tumors.

## 1 Introduction

*Helicobacter pylori* (*H. pylori*) is a gram-negative, spiral-shaped bacterium that specifically colonizes gastric epithelial cells. It is one of the most common bacterial infections in the world, affecting the gastric mucosa in approximately 50% of the world’s population (1, 2). *H. pylori* is a well-known risk factor of gastrointestinal disorders including chronic gastritis, peptic ulcer, functional dyspepsia and gastric cancer (3–5). It can induce chronic non-atrophic gastritis, and then progress to intestinal metaplasia and abnormal hyperplasia, and may ultimately develop into gastric cancer. Since 1994, it has been listed as a first-class carcinogen by the World Health Organization. Besides, recent studies have indicated that *H. pylori* is closely related to the development of some extra-gastric cancers. For example, Ralser A et al. proved that *H. pylori* is a strong pathogenic factor in the development of colorectal cancer (6). Pulikonda Mounika observed that patients infected with *H. pylori* have an increased risk of lung cancer (7). The mechanisms have yet to be clarified.

Different from conventional chemotherapy and radiotherapy, cancer immunotherapy is an emerging cancer treatment aiming to elicit an anti-tumor immune response. It can activate immune cells and enhance the ability of the immune system, control and kill cancer cells, and eventually cure cancer (8). Immunotherapy can actually date back to 1891 when William Coley noticed that mixtures of live and inactivated *Streptococcus pyogenes* and *Serratia marcescens* could cause tumor regression in sarcoma patients (9), and then first attempted to leverage the immune system to treat cancer. In recent years, cancer treatment has been revolutionized by the advent of immunotherapies, including cancer vaccines, immune checkpoint inhibitors (ICIs), adoptive immune cell therapy, cytokine therapy, and so on. Although some treatments like adoptive cell metastases and ICIs have demonstrated clinical efficacy, their application still remains limited, benefiting only a subset of cancer patients (10). The factors affecting the efficacy of immunotherapy have prompted comprehensive investigations recently.

Tumor micro-environment (TME) is composed of a variety of immune cells, signaling molecules and extracellular matrix components, whose overall state is significantly influenced by tumor cells, and in turn modulates the activity of tumor cells. The gut microbiota constitutes the most extensive and intricate microecosystem in the human body. It can influence TME by secreting small molecule metabolites that can spread from the initial location gut, and modulate both local and systemic anti-tumor immune responses (11). *H. pylori* can change the cells composition of TME by various virulence factors, such as vacuolating cytotoxin A(VacA), cytotoxin-associated gene A(CagA), and neutrophil activating protein (NAP). Then immune system status and the efficacy of immunotherapy should be affected (12).

Previously, research on the relationship between *H. pylori* and the efficacy of tumor immunotherapy focused on gastric cancer. An increasing number of studies now point out that *H. pylori* is also closely related to some extra-gastric cancers. *H. pylori* infection may have different effects on the efficacy of immunotherapy for cancers such as melanoma, colorectal cancer and non-small cell lung cancer (13–15).

Tumor immunotherapy, particularly immune checkpoint inhibitors, has been widely utilized in clinical treatment. However, its efficacy is affected by numerous factors, which restricts its promotion. A meta-analysis and systematic review of Gong et al. systematically analyzed the effect of *H. pylori* on tumor immunotherapy, concluded that *H. pylori* would reduce its efficacy, and suggested the underlying mechanisms (16). To understand the effects and the mechanisms of *H. pylori* on the efficacy of cancer immunotherapy more comprehensively, we summarized relevant literature recent years. In this review, we show that the effect of *H. pylori* on the efficacy of cancer immunotherapy is controversial, involving the evidence and mechanisms of both beneficial and adverse effects. In addition, we classify according to different cancer types, which can more clearly reflect the characteristics of systemic manifestations caused by *H. pylori* infection, and arouse people’s attention to the impact of *H. pylori* when performing tumor immunotherapy. Through a literature search, we identified four types of cancer: gastric cancer, colorectal cancer, non-small cell lung cancer and melanoma. They are closely related to *H. pylori*, and there are many articles that have examined the effect of *H. pylori* on the efficacy of their immunotherapy, so the review will focus on these four cancers.

## 2 The effects and mechanisms of *H. pylori* on cancer immunotherapy

### 2.1 Gastric cancer

#### 2.1.1 *H. pylori* and gastric cancer

*H. pylori* colonizes the gastric mucosa and plays an important role in the occurrence and development of gastric cancer(GC). *H. pylori* can regulate tumor progression by affecting various cells in the TME, such as tumor-associated macrophages, bone marrow mesenchymal stem cells, cancer-associated fibroblasts, and myeloid-derived suppressor cells (12). *H. pylori* can regulate tumor-associated macrophage differentiation, disrupt M1/M2(or Mreg) balance, and favor the M2 phenotype to evade immune surveillance (17, 18). *H. pylori* infection regulates macrophage function by modulating specific microRNAs (19, 20), such as upregulating the expression levels of let-7i-5p, miR-146b-5p, and miR-185-5p (21, 22). In addition, *H. pylori* may induce the expression of enzymes in macrophages, such as arginase 2 and matrix metalloproteinase, leading to immune evasion (23–25). Chronic inflammation caused by *H. pylori* infection recruits bone marrow-derived mesenchymal stem cells to stomach, which then undergo malignant transformation because of the clonal dominance. Bone marrow-derived mesenchymal stem cells can also decrease the proportion of T cells that produces IFN-γ and inhibit the proliferation of CD4+ and CD8+ T cells, leading to local and systemic immunosuppression and the development of *H. pylori*-induced GC (26).

Studies have shown that *H. pylori* infection upregulates the expression of programmed death protein ligand-1(PD-L1) in gastric mucosa cells, which then affects the prognosis of GC patients immunotherapy (27). Sonic hedgehog(SHH) pathway is considered to be involved in PD-L1 upregulation by *H. pylori* infection (28). *H. pylori* virulence factors play critical roles in immune regulation, such as type IV secretion system(T4SS) components that activates the p38 mitogen-activated protein kinase pathway and upregulates PD-L1 expression, thereby inhibiting T cell proliferation (29). Wang et al. pointed out that *H. pylori* CagA can upregulate the level of exosomal PD-L1 protein in *H. pylori*-infected GC through the CagA-p53-miR34a-PD-L1 signaling axis, which inhibits CD8+ T cells proliferation and cytokine secretion (30, 31).

Small extracellular vesicles(sEVs) from *H. pylori*-positive GC cells have been reported to promote lymphangiogenesis and lymphatic remodeling via transferring over-expressed miR-1246 in sEVs to lymphatic endothelial cells (LECs). Then it can lead to hymphatic genesis, stabilize PD-L1 which inducing CD8+T cell apoptosis (32). In conclusion, *H. pylori* infection can affect the TME, T cell functions and PD-L1 expression of gastric mucosa, which indicates the potential effects of *H. pylori* infection on immunotherapy.

In addition to gastric adenocarcinoma, there is also a strong association between *H. pylori* infection and gastric MALT lymphoma. Data show that about 80-90% of patients with gastric MALT lymphoma are infected with *H. pylori*, of which 60-80% can be relieved by eradication of *H. pylori* alone (33). Much evidence suggests that most patients with gastric MALT lymphoma achieve complete remission after *H. pylori* eradication (34–36). Therefore, *H. pylori* eradication is important in the management of gastric MALT lymphoma.

In addition to having an impact on the treatment of lymphoma, *H. pylori* infection increases the risk of gastric adenocarcinoma, although cases of primary gastric lymphoma (PGL) complicated by gastric adenocarcinoma are clinically rare. Amiot et al. observed a significantly increased risk of developing a second primary malignancy in patients with gastric MALT lymphoma treated with immunotherapy/chemotherapy, while the incidence was not high in patients receiving *H. pylori* eradication therapy alone (37). Another study has identified an increased risk of metachronous gastric adenocarcinoma in PGL patients. It may be attributed to factors such as *H. pylori* infection that exacerbates the damage to the gastric mucosa (38). This potential carcinogenesis promotion deserves attention.

#### 2.1.2 *H. pylori* affects the efficacy of immunotherapy for gastric cancer

Several studies have observed that *H. pylori* infection is related to the efficacy of immunotherapy in GC patients. In metastatic GC patients treated with ICI, *H. pylori* infection was associated with a lower survival (39). Similarly, in another analysis of patients with advanced GC, *H. pylori*-positive group had a higher risk of nonclinical response to anti-PD-1 therapy. The *H. pylori*-negative group had a longer overall survival (OS) and progression-free survival (PFS) than the positive group. Multivariate analysis indicated that *H. pylori* infection was independently associated with PFS, suggesting *H. pylori* infection is associated with the worse outcome of immunotherapy for advanced GC patients (40). However, some previous studies have come to the opposite conclusion. According to the result of randomized KEYNOTE-062 trial, the large international CheckMate-649 trial and the randomized ATTRACTION-4 trial, Asian patients exhibited better clinical benefits from anti-PD-1/PD-L1 therapy compared with North American and European patients, which is most likely related to a higher rate of *H. pylori* infection in Asian GC patients (41). And in KEYNOTE-062 trial, pembrolizumab demonstrated clinically meaningful efficacy in patients with a CPS of 10 or higher for PD-L1, with better survival in patients with high PD-L1 expression, indicating that patients with higher levels of PD-L1 expression showed a trend toward better survival (42, 43). A 2021 meta-analysis involving 1870 patients has shown that *H. pylori* infection was associated with tumor PD-L1 expression, suggesting that *H. pylori* infection may be a predictor of a favorable response to immunotherapy in GC (44). Another large retrospective study of 10,122 patients with different cancer types in 2024 found that in patients with Epstein-Barr virus-negative microsatellite-stable GC, the *H. pylori*-positive group had longer immune-related progression-free survival (irPFS) and immune-related overall survival(irOS) than the *H. pylori*-negative group. And *H. pylori*-positive patients had higher PD-L1 levels and more unexhausted CD8+ T cells, suggesting that *H. pylori* infection is a beneficial factor in GC immunotherapy by shaping the thermal tumor microenvironment (15).

Therefore, there is still controversy about the specific effect of *H. pylori* infection on the efficacy of immunotherapy for GC, and a larger clinical study is needed to confirm the association between them. Meanwhile, the mechanisms of *H. pylori* affecting the efficacy of immunotherapy for gastric cancer are also being studied.

Infiltrated by a variety of immune cells, TME plays a key role in the immune evasion of tumors by mediating immune tolerance. Therefore, TME-based immunotherapy demonstrates great potential for therapeutic development. Among them, ICIs have attracted extensive attention. It mainly includes two types, anti-cytotoxic lymphocyte-associated protein 4 therapy and anti-PD-1/PD-L1 therapy. At present, ICIs targeting the PD-1/PD-L1 signaling pathway have been applied to patients with various tumors, and have shown good therapeutic effects in malignant melanoma and non-small cell lung cancer (45). However, the efficacy of PD-1 and PD-L1 inhibitors for GC remains controversial (13, 46).

PD-1 and PD-L1 are both transmembrane proteins. As an important immunosuppressive molecule, PD-1 is the receptor of PD-L1 and is mainly expressed in activated T cells, B cells, activated monocytes, natural killer T cells, and dendritic cells (DCs). PD-L1 is more widely expressed than PD-1, appearing on human activated T cells, DCs, monocytes, and some non-hematopoietic cells(e.g., lung cells, vascular endothelium, liver nonparenchymal cells, placental synctiotrophoblasts, and keratinocytes) (47). When PD-L1 binds to PD-1, programmed death of T cells can be initiated. Notably, PD-L1 is also expressed in tumor cells. Therefore, when PD-1 on the surface of activated T cells binds to PD-L1 expressed by tumor cells, the activation, proliferation, survival, cytokine secretion, and cytotoxic activity of T cells are inhibited, resulting in impaired downstream anti-tumor immune responses and ultimately tumor cells immune escape (48). Therefore, the regulation of PD-1 and PD-L1 expression by *H. pylori* infection may affect the efficacy of checkpoint inhibitors targeting the PD-1/PD-L1 signaling pathway.

Some studies have suggested that up-regulation of PD-L1 expression by *H. pylori* will weaken the efficacy of tumor immunotherapy. The SHH pathway activated by *H. pylori* infection induces the expression of PD-L1 and the proliferation of tumor cells in GC, leading to the resistance to immunotherapy (12). GLI1 is a key transcription factor in the SHH pathway, and *H. pylori* infection reduces the methylation level of the GLI1 promoter region in an m6A-dependent manner, thereby activating the expression of GLI1 and promoting tumor proliferation (49). In addition, the effect of *H. pylori* upregulating PD-L1 expression is manifested in many cells, such as parietal cells, macrophages, eosinophils, and dendritic cells. Upregulated PD-L1 on these cells competitively bind to PD-1 on T cells, thereby inhibiting tumor killing by T cells, reducing the efficacy of anti-PD-1/PD-L1 therapy (50).

It has been suggested that increased PD-L1 expression might be a favorable factor for ICI therapy in GC (41). CagA is an important virulence factor encoded by *H. pylori*, and is closely related to the pathogenesis of gastric mucosa (51, 52). The CagA protein is mediated by the *H. pylori* T4SS and injected into the host gastric epithelial cells. Once it enters the cells, CagA can interact with multiple molecules, disrupt the normal signaling pathway, and cause cytopathy and transformation (53, 54). In addition, CagA is associated with the immune evasion by promoting the expression of PD-L1 in tumor tissues and exosomes via the p53-miR-34a-PD-L1 signaling axis (30). And *H. pylori* infection induced an increase in SHH and signaling via a CagA-dependent pathway, and elevated Shh signaling mediated *H. pylori*-induced PD-L1 expression (28). PD-L1 blockade can restore the proliferation and cytokine secretion of CD8+ T cells inhibited by CagA, which is a promising method to improve the efficacy of immunotherapy for *H. pylori*-infected GC (30). Recently, other factors regulating PD-L1 in *H. pylori*-infected gastric epithelial cells has also been studied. Among them, miR-140 can directly inhibit PD-L1 expression. In *H. pylori*-positive GC, miR-140 expression is significantly reduced and PD-L1 expression is significantly increased. It has been suggested that *H. pylori* infection may influence the efficacy of anti-PD-1/PD-L1 therapy by decreasing the expression level of miR-140 and thereby increasing the level of PD-L1 in cancer cells (55).

In addition to regulating PD-L1 expression, the potential immunotherapeutic value of *H. pylori* vaccine-activated CD3+ T cells in patients with advanced GC was also revealed. The *H. pylori* DNA vaccine induces a shift from Th1 to Th2, which mimics the immune status of GC patients with chronic *H. pylori* infection. Stimulated CD3+ T cells inhibit the growth of human GC cells *in vitro*, and adoptive transfusion of CD3+ T cells inhibits the growth of GC xenografts *in vivo*, suggesting the anticancer effects of CD3+ T cells both *in vitro* and *in vivo*. This may be due to a greater proportion of CD8+/CD4+ T cell infiltration, decreased regulatory FOXP3+ T cell infiltration, upregulation of Caspase-9/Caspase-3, and enhanced apoptosis induced by downregulation of Survivin (56). Taken together, increased PD-L1 expression and CD3+ T cells may be favorable factors for ICI therapy for GC. Therefore, it is speculated that *H. pylori* infection may be a novel biological factor that may affect the survival benefit of patients with advanced GC treated with first-line ICIs (41).

### 2.2 Colorectal cancer

#### 2.2.1 *H. pylori* and colorectal cancer

Although *H. pylori* typically colonizes the gastric mucosa and primarily targets the stomach, there are increasing evidences demonstrating the association between *H. pylori* infection and other extra-gastric diseases, including colorectal cancer (CRC). Data showed that *H. pylori* infection was associated with a higher risk of colorectal adenoma and CRC, with pooled odds ratios of 1.51 and 1.70, respectively (57, 58). A study of 47,714,750 patients demonstrated an independent positive correlation between CRC risk and history of *H. pylori* infection (59). Another retrospective study of 96,572 subjects who had been eradicated from *H. pylori* concluded that the incidence of CRC gradually decreased to a lower level than that of the general population, especially rectal cancer (60). Together, these evidences suggested a strong link between *H. pylori* and CRC.

A number of studies have also confirmed that *H. pylori* infection can directly promote the development of CRC *in vivo* and *in vitro*. In an employed Apc mutant mouse lines(Apc^+/min^ and Apc^+/1638N^) as a surrogate model of human CRC, an almost twofold increase in the number of tumors in mice infected with *H. pylori* was observed, which is consistent with the fact observed in epidemiology that people infected with *H. pylori* have a nearly twofold increased risk of developing CRC (6). The pro-inflammatory Th17 response induced by *H. pylori*, especially the differentiation of Treg cells into the Th17 phenotype, may constitute one of the main mechanisms for enhancing tumor development. In addition, the strong pro-inflammatory response induced locally by *H. pylori* in the small intestine is accompanied by the activation of NF-κB and STAT3 pathways. Activated STAT3 levels in tissues are also related to reduced tumor invasion, tumor, lymph node, metastatic stage, and overall survival in CRC patients (61). *H. pylori* virulence factors may also affect the development and progression of CRC. CagA increases the levels of the pro-survival factor phosphorylated ERK and the anti-apoptotic protein MCL1, therefore dampening gut epithelial self-renewal by inhibiting apoptosis (62). In addition, *H. pylori*(CagA+) inhibits the expression of miR-125b-5p in CRC cells and promotes autophagy, contributing to the development and invasion of colon cancer (63). Another virulence factor VacA enters cells and mitochondrial membranes through the formation of chloride(Cl-) channels, resulting in loss of mitochondrial membrane potential, mitochondrial fragmentation, reactive oxygen species formation, autophagy (64). Cl-channel abnormalities are associated with cystic fibrosis, and cystic fibrosis is known to be associated with CRC. Hence, it is suggested that VacA-induced Cl-channel abnormality may be associated with CRC (65).

*H. pylori* can also affect CRC through indirect pathways. *H. pylori* is known to affect the local gastric microbiota as well as distant microbiota in the intestine and colon (66). Inflammation-driven intestinal dysbiosis microbiota can promote the formation and progression of colorectal tumors (67). Enrichment of the genera *Akkermansia* and *Ruminococcus* was found, and there was an inverse correlation between the presence of *Akkermansia* and gastrointestinal disease (68). *H. pylori* infection destroys intestinal mucus integrity in mice models, and a general loss of goblet cells were also observed, which are important for the production of mucin and antimicrobial peptides. The over-expression of certain mucins in tumors is likely to be the reason for the increase in *Akkermansia* in CRC patients (69). In all, *H. pylori* infection promotes CRC by altering intestinal homeostasis, including the integrity of intestinal mucosa, the immune barrier, and intestinal microbiota.

In addition, *H. pylori* can alter gastrin levels in the body and may have an effect on CRC. *H. pylori* gastritis can cause atrophic gastritis and reduce gastric acid secretion. As a result, the concentration of gastrin in the blood increases. Gastrin causes overgrowth of the large intestine mucosa and is strongly associated with the development of colorectal tumors (70–72). The over-expression of gastrin and gastrin receptors(CCKBR) and cyclooxygenase-2(COX-2) may stimulate tumor growth, angiogenesis, and reduce apoptosis (73). Therefore, gastrin may be a potential factor in the development of CRC in *H. pylori* infection (74). However, there is still much controversy about whether *H. pylori* contributes to CRC (75, 76) and further studies are needed to confirm the association.

#### 2.2.2 *H. pylori* affects the efficacy of immunotherapy for colorectal cancer

The traditional treatment methods for CRC are mainly radiotherapy, surgical resection, etc. However, CRC is difficult to detect in the early stage. When patients show clinical symptoms, it is often at an advanced stage and metastasize to distant places, and traditional treatments are not effective at this time (77). Fortunately, ICI-based immunotherapies in patients with CRC have achieved satisfactory results in terms of safety and efficacy. Multiple immunotherapy drugs, including pembrolizumab, nivolumab, and ipilimumab, have been approved for the treatment of advanced CRC. At the same time, new therapies such as CAR-T and cancer vaccines also have good prospects (78). Therefore, elucidating the factors influencing immunotherapy for CRC will benefit more patients.

In mice treated with anti-CTLA4 alone, the tumor volume of *H. pylori* infected mice was significantly larger than that of uninfected mice. Same results were observed in mice treated with a combination of anti-CTLA4 and anti-PD-L1. It can be concluded that mice infected with *H. pylori* are less sensitive and responsive to ICIs. In addition, the number of colon tumors in uninfected mice treated with anti-CTLA4 was significantly lower than in *H. pylori* infected mice (14). Therefore, it is suggested that *H. pylori* infection appears to play a disruptive role in immunotherapy for CRC.

Clinical data also support that *H. pylori* has an adverse effect on ICIs for CRC. DNA mismatch repair-deficient(dMMR)/microsatellite instability-high(MSI-H) colorectal adenocarcinoma has a high sensitivity to immunotherapy and has been approved for anti-PD-1/PD-L1 immunotherapy. Based on data from patients with dMMR/MSI-H colorectal adenocarcinoma who received anti-PD-1/PD-L1 immunotherapy, researchers found that the median irPFS of patients with *H. pylori*-positive cancer was significantly shorter than that of *H. pylori*-negative patients, and the same discrepancy were observed in irOS. In addition, there is also a trend towards a shortening of irFPS in *H. pylori*-positive patients with dMMR/MSS colorectal adenocarcinoma (15). These data indicate that *H. pylori* may reduce the effectiveness of ICIs and is not favorable to patient outcomes.

In conclusion, it has been observed that *H. pylori* infection can diminish the efficacy of immunotherapy of CRC. Therefore, before administering immunotherapy to patients with CRC, it may be advisable to detect and eradicate *H. pylori* to attenuate its negative influence on immunotherapy.

### 2.3 Non-small cell lung cancer

#### 2.3.1 *H. pylori* and NSCLC

*H. pylori* infection may be relevant to the risk of lung cancer, but the evidence supporting this association remains controversial due to variations in sample size, sample selection criteria, and research methodology. Some data have shown that *H. pylori* biomarkers, such as VacA, CagA, *H. pylori*1564, and catalase, were significantly associated with an increased risk of lung cancer (79). Multiple meta-analysis show that patients with *H. pylori* infection were observed a remarkably increased risk of lung cancer (7, 80). Seroprevalence of *H. pylori* antibody in lung cancer patients was significantly higher than that in non-lung cancer patients (81). The seroprevalence of *H. pylori*, especially that expressing CagA, is significantly higher in lung cancers than in healthy controls (82).

Gastric reflux containing *H. pylori* components through the oral-pharyngeal-laryngeal axis may lead to respiratory colonization of *H. pylori (* 83). *H. pylori* can also be detected in dental plaque and saliva in human subjects, suggesting that the oral cavity may be an extra-gastric repository for *H. pylori (* 84), which can lead to inflammation of the airways and the development of lung cancer. In fact, the presence of *H. pylori* and/or its virulence factors in the mucous membranes of the lungs and upper respiratory tract has been identified (83). VacA was identified in surgical lung biopsy tissue samples from patients with pneumonia (85). Inhaled into the lungs, VacA can stimulate lung epithelial cells to secrete inflammatory cytokines and damage lung tissue. Moreover, *H. pylori* urease protein has also been found to enter the lungs through gastroesophageal reflux disease, providing an antigenic trigger for the development of pulmonary granulomas contributing to proliferation and carcinogenesis of the lung mucosa (83, 86).

Increased gastrin concentrations caused by *H. pylori* in the stomach may also stimulate lung cell proliferation. In fact, gastrin levels are elevated in lung cancer tissues (82). Studies have shown that *H. pylori* infection in lung cancer patients is accompanied by significantly elevated gastrin levels in plasma and tumor tissues, as well as increased cyclooxygenase-1(COX-1) and cyclooxygenase-2(COX-2) expression (82). COX-1 and COX-2 are the critical enzymes for the production of prostaglandin E2(PGE2), and the COX-2 has been found overexpressed in many cancers. PGE2 is a prostanoid lipid associated with enhancement of cancer cell survival, growth, migration, invasion, angiogenesis, and immunosuppression (87). Gastrin may contribute to lung cancer by inducing bronchial epithelial mucosal cell proliferation, atrophy, and induction of COX-2. In addition, lipopolysaccharide of *H. pylori*, may stimulate the production of proinflammatory cytokines like interleukins (7). Chronic inflammatory irritation resulting from this may also be the cause of lung cancer.

In addition, the signaling adaptor protein p130^Cas^ in tumor cell is associated with carcinogenesis and prognosis of lung cancer. Specifically, p130^Cas^ over-expression has been linked to reduced OS in lung cancer patients (88, 89). Therefore, p130^Cas^, activated by Src kinase following *H. pylori* infection, may involve in lung carcinogenesis during *H. pylori* infection (83).

#### 2.3.2 *H. pylori* affects the efficacy of immunotherapy for NSCLC

In addition to influencing the occurrence of NSCLC through direct or indirect pathways, *H. pylori* infection may also be associated with the efficacy of immunotherapy for NSCLC.

The treatment of NSCLC was mainly surgery, radiotherapy, and chemotherapy for an extended period. However, recent studies have shown that NSCLC is one of the most sensitive cancers to ICIs. Many studies have demonstrated that ICIs, including atezolizumab, pembrolizumab and nivolumab, exhibit good efficacy in the treatment of NSCLC (90–92). However, some patients exhibit resistance to immunotherapy. Therefore, it is essential to identify the factors that affect patient prognosis in order to benefit NSCLC patients.

In a cohort of 60(18 *H. pylori* serology-positive) NSCLC patients in France, *H. pylori* seropositivity was associated with a significant decrease in survival of NSCLC patients after receiving anti-PD-1 therapy(p = 0.0001) with median survival of 6.7 months and 15.4 months, respectively. A decline in OS was also noted in a cohort of 29 patients(9 *H. pylori* serology-positive) in Canada(9.3 versus 21.7 months) and PFS was also observed among patients with *H. pylori*-positive NSCLC. These results showed that *H. pylori* serology positivity was associated with poor efficacy of anti-PD-1 therapy (14). In addition, the number of monocyte cell lines in *H. pylori*-positive tumors was significantly reduced compared to *H. pylori*-negative patients. Additionally, the gene expression induced by type I interferon, IFN-γ, and IL-6 was also decreased. The decrease in monocytes indicates that inhibition of the host innate immune response induced by *H. pylori* may reduce the efficacy of cancer immunotherapy (14). This may be a mechanism by which *H. pylori* affects tumor immunotherapy. Further study also confirmed that the reduced efficacy of tumor immunotherapy with *H. pylori* infection is independent of alterations in the fecal microbiota caused by *H. pylori (* 13).

Up to now, there are still few studies investigating the effect of *H. pylori* on the efficacy of immunotherapy in NSCLC patients. Existing studies have limitations such as inadequate sample size and *H. pylori* detection methods, and more comprehensive studies are needed to fill the gaps (46).

### 2.4 Melanoma

#### 2.4.1 *H. pylori* and melanoma

Melanoma is one of the most aggressive forms of skin cancer that develops from skin melanocytes located in the basal layer of the epidermis. Ultraviolet radiation is the main cause of cutaneous melanoma (93). Over the past years, there has been a rise in the incidence of melanoma and melanoma-related mortality, which poses a threat to the healthcare system (94). Research on the relationship between *H. pylori* and melanoma has mainly focused on the application of *H. pylori* neutrophil activating protein (HP-NAP) in the treatment of melanoma. HP-NAP is a dodecameric protein that stimulates the maturation and differentiation of monocytes and immature dendritic cells. These cells subsequently produce cytokines such as tumor necrosis factor-α(TNF-α), IL-6 and IL-12 (95), which regulate the immune response and potentially influence tumor development. Recent studies have observed that HP-NAP effectively inhibited the growth and metastasis of melanoma and improved the OS rate of zebrafish of melanoma xenograft, which was accompanied by strong recruitment of macrophages with a pro-inflammatory profile at the tumor site (96). In addition, recombinant *H. pylori* neutrophil activation protein(rMBP-NAP) prevented lung metastasis from melanoma and induced systemic Th1 immune response with anti-angiogenic activity (97). It was also proved that rHP-NAP promoted the maturation of dendritic cell vaccine and enhanced the cytotoxic response. rHP-NAP may serve as an adjuvant against melanoma dendritic cell vaccines (98).

In conclusion, the NAP induced by *H. pylori* may have a promising future in the treatment to melanoma.

#### 2.4.2 *H. pylori* affects the efficacy of immunotherapy for melanoma

Melanoma is highly sensitive to immunotherapy attributed to its elevated tumor mutational load and high T cell infiltration. In 2011, ipilimumab, the first CTLA-4 immune checkpoint inhibitor, was approved for the treatment of advanced inoperable metastatic melanoma. In the following ten years, multiple monoclonal antibodies have been used to treat melanoma at different stages, such as nivolumab and pembrolizumab, which have achieved good efficacy and significantly improved the survival rate of patients with advanced melanoma (99). Despite advancements, some melanoma patients treated with immunotherapy continue to encounter recurrence and adverse effects (100). Therefore, it is crucial to identity the specific factors that affect their efficacy. Among these factors, the influence of *H. pylori* has attracted widespread attention.

*H. pylori* infection may hinder the immunotherapy response in melanoma. Compared with *H. pylori*-negative patients, *H. pylori*-positive patients with melanoma had significantly shorter OS(p = 0.02), PFS and objective response rate, suggesting that *H. pylori* infection affected the efficacy of ICIs (13). In addition, in B16-OVA melanoma model, mice were injected with CpG-adjuvanted OVA peptide, which served as a cancer vaccine. Notably, the tumor volume in *H. pylori*-uninfected vaccinated mice was significantly smaller than that of *H. pylori*-infected vaccinated mice. This indicated that the vaccine efficacy was diminished in the presence of *H. pylori*-positive tumor-bearing mice (14).

The mechanism that *H. pylori* diminishes the efficacy of melanoma immunotherapy is not yet well understood. But it is certain that it’s independent of the alteration in the fecal microbiota composition induced by *H. pylori (* 13, 14). There were no significant differences in the alpha diversity metrics of fecal microbiota in *H. pylori*-positive or negative patients. And beta diversity analysis between both groups showed no significant cluster difference following statistical analysis. This indicates that the presence or absence of *H. pylori* infection does not affect the overall diversity of the human fecal microbiota. Furthermore, the presence of *parahaemodhemostreptococcus* was associated to resistance against ICIs in melanoma (13). Transplanting the feces of *H. pylori*-positive mice to *H. pylori*-negative mice did not reduce the efficacy of cancer vaccines, while transplanting feces from *H. pylori*-negative mice to positive mice still reduced the efficacy of cancer vaccines (14). More research is needed to elucidate the underlying mechanisms.

In conclusion, *H. pylori* weakens the efficacy of immunotherapy in experimental models and clinical patients, but the specific mechanisms remain unclear. *H. pylori* infection should be taken into account in the treatment of melanoma.

## 3 Summary

**Figure 1:**
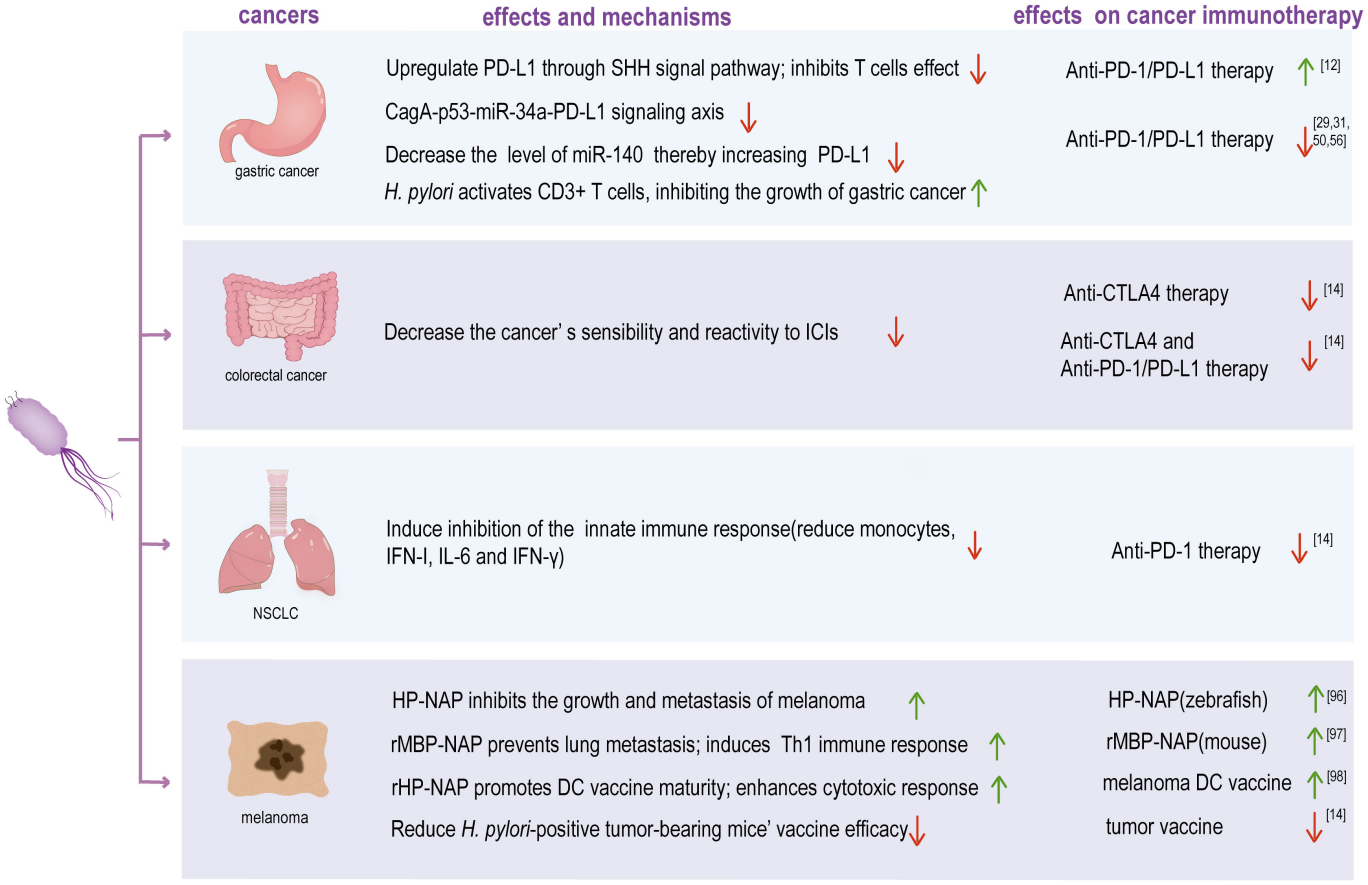
The effect of *H. pylori* infection on the efficacy of immunotherapy for various cancers and potential mechanisms. NSCLC, non-small cell lung cancer; CagA, cytotoxin-associated gene A; SHH signal pathway, Sonic hedgehog signal pathway; PD-L1, programmed death ligand 1; PD-1, programmed cell death protein 1; CTLA4, cytotoxic T lymphocyte-associated antigen-4; ICIs, immune checkpoint inhibitors; IL-6, interleukin 6; IFN-γ, interferon-γ; HP-NAP, *H. pylori* neutrophil activating protein; rMBP-NAP, recombinant HP-NAP with the maltose-binding protein of *Escherichia coli*; rHP-NAP, recombinant *H. pylori* neutrophil activating protein; DC, dendritic cell. The green arrow indicates a positive effect on tumor immunotherapy, and the red arrow indicates a negative effect on tumor immunotherapy.

This review summarizes current research progress on the effects of *H. pylori* on the cancer’s development and immunotherapy (**Figure 1**). Immunotherapy for oncology is developing rapidly and is becoming a new option for patients who can’t benefit from traditional therapies. However, immunotherapy is limited until now. There are many patients who do not respond well to tumor immunotherapy. Identifying the factors affecting the efficacy of immunotherapy is crucial for improving immunotherapy efficacy. Studies have found that *H. pylori* infection plays a role in both intragastric and extra-gastric diseases, and even has a strong impact on tumor immunotherapy. Numerous clinical investigations have shown that the responsiveness to immunotherapy is related to *H. pylori*-positive status, such as GC, CRC, NSCLC, and melanoma. The effects of *H. pylori* on patients with different cancer types may be different, and the mechanisms may be related to the activation on immune cell status, PD-L1 expression level regulation, inflammatory pathway action and so on (6, 7, 13, 39, 101). Based on different effects of *H. pylori* on different cancers, whether to eradicate *H. pylori* may be an important consideration in clinical practice to improve immunotherapy efficacy (33, 60). On the contrary, utilizing *H. pylori* to improve the efficacy of immunotherapy, such as the application of HP-NAP in the treatment of melanoma, may be a promising application (96, 97, 102).

In the future, more clinical data and studies are needed to ensure the role of *H. pylori* in immunotherapy. This will facilitate the identification of more specific mechanisms and targets to effectively select patients who are likely to benefit from immunotherapy, thereby offering insights for the research and development of novel therapeutics.
